# Pilot-scale study on catalytic ozonation of bio-treated dyeing and finishing wastewater using recycled waste iron shavings as a catalyst

**DOI:** 10.1038/s41598-018-25761-6

**Published:** 2018-05-15

**Authors:** Jieting Ma, Yunlu Chen, Jianxin Nie, Luming Ma, Yuanxing Huang, Liang Li, Yan Liu, Zhigang Guo

**Affiliations:** 1 Department of Environmental Science and Engineering, Fudan University, Shanghai, 200433 China; 2 College of Environmental Science and Engineering, Tongji University, Shanghai, 200092 China; 3 School of Environment and Architecture, University of Shanghai for Science and Technology, Shanghai, 200093 China

## Abstract

A pilot scale reactor with an effective volume of 2.93 m^3^ was built *in-situ* and run in both batch and continuous modes to investigate the removal for organic pollutants in bio-treated dyeing and finishing wastewater by heterogeneous catalytic ozonation under neutral pH with waste iron shavings as a catalyst. Experimental results showed that both running modes were able to reduce the chemical oxygen demand (COD) from 132–148 mg/L to a level below the discharge criteria (<80 mg/L) within 15–30 mins under several conditions. Specifically, significantly organic removal was observed with COD, soluble COD (sCOD) and dissolved organic carbon (DOC) decreased from the initial 165, 93 and 76 mg/L to 54, 28 and 16 mg/L respectively, when treated by 10.2 g-O_3_/min of ozone dosage at a hydraulic retention time of 30 mins under continuous mode. 80% proteins and 85% polysaccharides were removed with a decrease in their contribution to sCOD from 69% to 43%. Mineralization as well as conversion of high molecular organic compounds was observed through Gas Chromatography-Mass Spectrometer (GC-MS) & Liquid Chromatography-Mass Spectrometer (LC-MS) analysis, which led to a decrease of inhibitory effect from 29% to 25%, suggesting a reduction in the acute toxicity.

## Introduction

The textile industry was characterized by its enormous water consumption and complexity of chemicals used. Dyestuffs, textile biocides, stain repellents, and flame retardants were typical contaminants in the dyeing and finishing wastewater (DFW)^1,2^. After biological treatment by different types of activated sludge processes, the effluent organic matters (EfOM) still consisted of many refractory compounds as well as large amounts of soluble microbial products (SMPs) like proteins and polysaccharides. In some cases, the biological process might even convert the parent compounds into more toxic metabolites^3^, which would bring threat to the natural aqueous environment. The latest discharge limitation of 80 mg/L COD for water pollutants from dyeing and finishing of textile industry (GB 4287-2012) leads to a challenge to the textile industry. Various methods including adsorption^4^, advanced oxidation processes (AOPs)^5–7^, membrane distillation^8^ and membrane bioreactors^9^ have been tried for the tertiary treatment of bio-treated dyeing and finishing wastewater (BDFW) to further remove EfOM.

AOPs involved the generation of highly reactive oxidative species such as hydroxyl radicals, superoxide radical anions, hydroperoxyl radicals, singlet and triplet oxygen, which showed great capability to degrade or mineralize bio-recalcitrant organic pollutants from contaminated wastewaters, thus AOPs were often implemented as additional advanced or tertiary treatment for wastewater treatment plants (WWTPs) prior to discharging into the environment^10–12^. Typically, ozone (O_3_) and hydrogen peroxide (H_2_O_2_) were the most frequently used oxidants in various techniques such as O_3_, UV/O_3_, O_3_/UV/H_2_O_2_, H_2_O_2_/UV^6^, Fe^2+^/H_2_O_2_, FeS/H_2_O_2_, etc.

Ozonation is an ideal technique due to the high oxidation potential of ozone (2.07 V), environment-friendly product of oxygen (O_2_) and low sludge production. Several reports elucidated the application of ozonation for further treatment of municipal or textile wastewater in pilot or full scale plants^13,14^. However, low efficiency was frequently observed for ozonation due to its selective oxidation and slow mineralization rates for organic compounds. Under this situation, catalytic ozonation was developed as one of the most promising AOPs^15^. Heterogeneous catalytic ozonation used catalysts like carbon materials, supported or unsupported metals and metal oxides, semiconductors, and natural minerals to trigger the generation of highly reactive species such as ·OH with a higher oxidation potential of 2.80 V^16–19^. Although heterogeneous catalytic ozonation had been proven to be effective in removing many bio-recalcitrant organic contaminants such as chloro nitrophenol^20^, polycyclic aromatic hydrocarbons^21^, atrazine^22^, etc., it was found mainly in the process of laboratory investigation. Pilot or full scale applications were rarely reported due to the instability and reduced efficiency of recycled catalysts.

Iron materials were frequently employed in the heterogeneous catalytic ozonation as catalysts, which have been used in various form of zero-valent iron (ZVI), iron oxides, alloys, iron-loaded on activated carbons or other supports^23–26^, and all showed great catalytic activity in ozonation system. Another possible reason for the wide use of iron catalyst is its low toxicity and cheap price compared with other transition metals. In our previous research^27^, waste iron shaving was proved to be an effective and stable catalyst for ozonation of BDFW. Low price, convenience of separation, effectiveness during successive runs all indicated the possibility of its application in pilot or full scale plants.

In this study, a pilot scale reactor using heterogeneous catalytic ozonation was designed and built *in-situ* to evaluate the possibility of treating BDFW from textile industry. The main objectives of this work were as follows: (1) Try to test if organic compounds in BDFW could be effectively removed so as to meet the requirement of GB 4287–2012; (2) Investigate both batch and continuous running modes, relative parameters and the corresponding organic removal efficiencies as well as the long term stability; (3) Explore the variation of different types of organic compounds in BDFW before and after treatment by heterogeneous catalytic ozonation with iron shavings as catalyst.

## Materials and Methods

### Materials and reagents

All the experimental studies were performed with real wastewater from a 600,000 m^3^/d dyeing and finishing industrial wastewater treatment plant located in southeast of China. A typical flow of hydrolysis acidification, aeration tank and secondary sedimentation was employed with a hydraulic retention time (HRT) of 3 h, 9 h and 3 h, respectively. Waste iron shavings was collected from a metal machinery plant using 38CrMoAl steel and pretreated by a special method patented with applying and opening numbers of CN105396590A and 2015107330134, respectively.

The chemicals used in this research were all of analytical grade or above, except for frequently used industrial pure PAM in real wastewater treatment plant. Sodium sulfite (Na_2_SO_3_), sodium nitrate (NaNO_3_), and potassium dihydrogen phosphate (KH_2_PO_4_) were purchased from Sinopharm Chemical Reagents Co. Ltd (Shanghai, China). The luminescent bacteria Photobacterium phosphoreum were bought from Hamamatsu Photon Techniques Inc., China.

### Experimental setup and procedures

**Figure 1:**
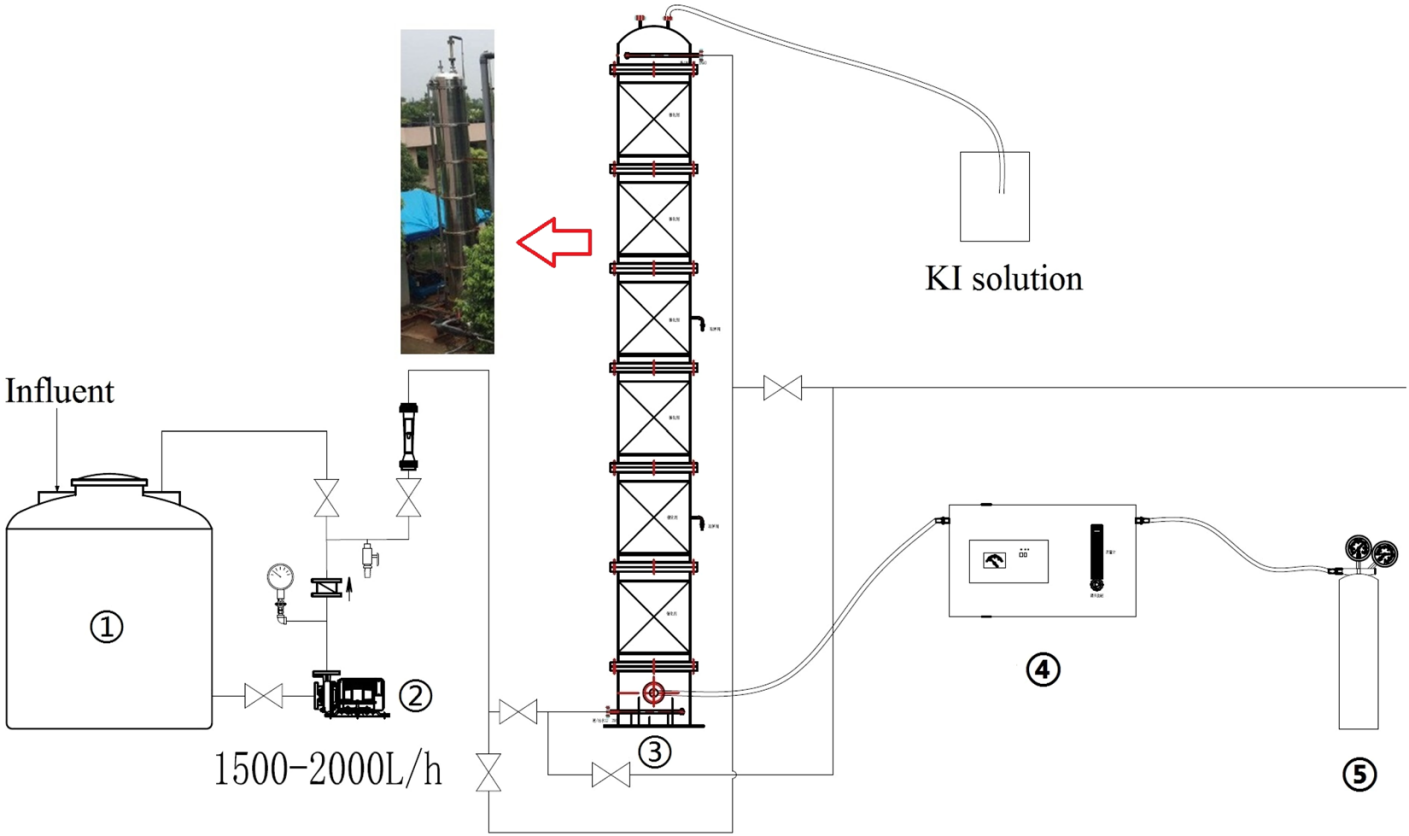
Pilot scale experimental set-up (1. storage tank; 2. influent pump; 3. reaction tower; 4. ozone generator; 5. oxygen tank).

The pilot scale setup was designed based on the characteristics of wastewater and experimental results obtained in the laboratory. As shown in Fig. 1, the O_3_/ZVI experiment was conducted in a six layer tower reactor with an inner diameter of 760 mm and effective height of 6450 mm, resulting in an effective volume of 2.93 m^3^. ZVI with a surface area of 2600 m^2^/m^3^ was pretreated and packed to a height of 910 mm into each layer, leading to a porosity of 95.5%. The secondary effluent of the wastewater treatment plant was fed directly into the reactor. Ozone gas was generated through a 1.5 kg O_3_/h corona discharge ozone generator (QRKY-MIOG-A-1.5, KINGWING Environmental Protection Co. Ltd., Beijing, China) fed with oxygen from a 7 m^3^/h oxygenator (PO-7-90, SINCE Gas System Co. Ltd., Suzhou, China). The gas mixture was continuously bubbled into the reactor through a porous plate. The concentration of ozone (80–160 mg/L) can be adjusted by the output power of the ozone generator, while the flow (1.2–2.0 m^3^/h) was regulated through the three-port valve. The off-gas was absorbed by the KI solution. The reactor was running in both batch and continuous modes. In the batch mode, 2.93 m^3^ of wastewater was fed into the reactor initially, and then circulated at a flow rate of 22 m^3^/h by a recirculating pump (WB70/055, Yuehua pump, Xinli Industrial Co. Ltd., Yangjiang City, China). In the continuous mode, a centrifugal pump (SZ037, Yuehua pump, Xinli Industrial Co. Ltd., Yangjiang City, China) was employed to feed the reactor at various influent flow rates of 2.9–17.6 m^3^/h to keep hydraulic retention time (HRT) within the range of 10–60 mins. The effluent was recycled at rates of 0–80 m^3^/h with the aid of recirculating pump. For those experiments about adding polyacrylamide (PAM), 2 mg/L PAM was added into influent of wastewater for this reactor, and the effluent then settled about 0.5 h to get a clear supernatant for further analysis.

The ozone utilization efficiencies (OUE) under different conditions were evaluated by COD reduction per unit ozone input (g ΔCOD/g ozone) by using equations (1) and (2) for batch and continuous modes, respectively.

1 $${\rm{Batsi}}\,\mathrm{mode}:\,OUE(g{\rm{\Delta }}COD/g{O}_{3})=\frac{({C}_{0}-{C}_{t})\times V}{{[{O}_{3}]}_{g}\times t\times {Q}_{g}}$$

2 $${\rm{Continuous}}\,{\rm{mode}}:\,OUE(g{\rm{\Delta }}COD/g{O}_{3})=\frac{({C}_{0}-{C}_{e})\times HRT\times {Q}_{l}}{{[{O}_{3}]}_{g}\times HRT\times {Q}_{g}}=\frac{({C}_{0}-{C}_{e})\times {Q}_{l}}{{[{O}_{3}]}_{g}\times {Q}_{g}}$$

In both equations (1) and (2), [O_3_]_g_ and Q_g_ represents O_3_ concentration (g O_3_/m^3^ gas) and flow (m^3^ O_3_/h) in influent gas for certain experiment. For equation (1), C_0_ & C_t_ represents COD value (mg/L) at zero and sampling time t (h), respectively. V equals to 2.93 m^3^. For equation (2), C_0_ & C_e_ represents COD value (mg/L) at influent and effluent of wastewater, respectively. Q_l_ is the inflow of the wastewater (m^3^ wastewater/h).

Samples were taken from the sampling port and quenched immediately with the addition of a suitable amount of sodium sulfite. The original sample was used for total iron and ferrous iron analysis. COD, acute toxicity, total phosphate were analyzed for supernatant after 30 mins sedimentation. The other analyses were performed after filtration with 0.45 µm membrane and proper dilution based on the upper limits of certain analysis method.

### Analytical methods

DOC and TN were analyzed with a TOC analyzer (Shimadzu TOC-L, Japan). pH and dissolved oxygen (DO) were determined with an instrument equipped with both pH (Sartorius PB-10,German) and DO probe (INESA JPB-607A, China), respectively. The COD, sCOD, total phosphorus (TP), Fe^2+^ and total Fe (TFe) were determined according to standard methods^28^. The ozone concentration in the gas phase was measured by the iodometric method^29^. The protein and polysaccharide concentrations were monitored by using the Lowry’s method^30^ and anthrone colorimetric assay^31^, respectively. The acute toxicity was evaluated following the procedure reported by Liu *et al*.^32^, to be brief, using the luminescent bacteria Photobacterium phosphoreum together with a toxicity analyzer (GR-8500A, Hangzhou Green Water Science & Technology CO., LTD, China). The variation of organic profiles in the wastewater was assessed by 3DEEM fluorescence, UHPLC-QTOF and GC-MS, respectively. The detailed conditions of fluorescence spectra and UHPLC-QTOF were listed in the supporting information. GC-MS (Shimadzu GCMS-QP2010 SE, Japan) analysis for organic compounds was performed with HP5-MS column according to USEPA method 625^33^.

### Data availability statement

All data are available based on request.

## Results and Discussion

### Catalytic ozonation performance under batch mode

**Figure 2:**
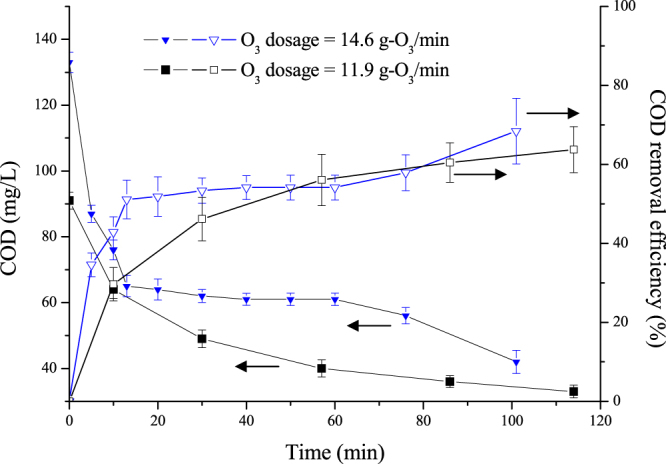
COD reduction during catalytic ozonation under batch mode. Experimental conditions: pH = 7; effective volume = 2.93 m^3^; recycling rate = 22 m^3^/h (ozone concentration = 120 mg/L; ozone flow rate = 7.3 & 5.84 m^3^-O_3_/h).

In this research, BDFW was treated by heterogeneous catalytic ozonation in batch mode under two conditions. The results in Fig. 2 show that the organic pollutants in BDFW were evidently removed by the catalytic ozonation processes. When the ozone dosage was 14.6 g-O_3_/min, the COD in the BDFW influent declined from the initial value of 133 mg/L to 65 mg/L in only 13 min, corresponded to the COD removal efficiency of 51%. The OUE can be calculated to be 1.14, 0.69, 0.47, 0.24 and 0.18 g COD/g ozone for the treatment time of 10, 20, 30, 60 and 101 mins, respectively. When the ozone dosage was 11.9 g-O_3_/min, the COD in the influent dropped from 91 mg/L to 64 mg/L in as short as 10 min and corresponded to a COD removal of 30%. The OUE was calculated to be 0.68, 0.35, 0.22 and 0.13 g COD/g ozone for the treatment time 10, 30, 57 and 114 mins, respectively. OUE was found to increase with the initial COD concentration but decrease with treatment time. Generally, higher ozone dosage brought to improved contaminant removal. Extension of reaction time was beneficial for pollutants removal but the removal rates began to slow down after 15 mins, especially for the former one. The reaction time of more than 100 min could elevate the COD removal percentage to around 65% in both experiments, and obtained a final COD of less than 40 mg/L, which was far below the criteria of 80 mg/L for direct discharge according to GB 4287-2012.

For the treatment of BDFW or textile wastewater, the required ZVI dosage was relatively higher than other types of catalysts. This might be explained by the high density of ZVI, which could not be mixed throughly with wastewater compared with those suspended or fluidized powder catalysts. Hydroxyl radical was generated on the active sites of catalysts with a life period of 10^−7^–10^−8^ s, which was too short to move in the aqueous system. In this situation, organic compounds typically reacted with hydroxyl radicals near the active site of catalysts. High dosage of ZVI provided large amount of active sites, which was beneficial for catalytic ozonation process. Pan *et al*. explored the effect of ZVI/ozone combination in degradation of bio-treated textile mill wastewater, and reported that under the condition of 150 L/h ozone, 600 g/L ZVI (particle size of 0.9–2 mm), the COD decreased from 127 mg/L to 43 mg/L in 3 h^34^. In another case, Polat *et al*. examined the heterogeneous catalytic ozonation of a textile wastewater in a continuous reactor used alumina as catalysts, they determined that the COD removal reached 26% in 20 min at pH 4 when the initial COD was 180 mg/L, with the ozone gas concentration being 43.2 mg/L and catalyst dosage being 60 mg/L^35^.

Actually in the field of heterogeneous catalytic ozonation, sole ZVI usually exhibited low efficiency alone near neutral pH, thus was frequently combined with other materials to enhance its catalytic activity, leading to a higher cost due to its complicated catalyst preparation procedure^26^. For example, pure ZVI micro-particles exhibited inert property with respect to ozone, and at a pH of 6.5, it increased the degradation of 10.3 mg/L ibuprofen by only approximately 3% during 1 h of treatment. At the same time, the combined ZVI-graphite improved the ibuprofen mineralization by 35%^36^. While for acid pH of 3, Martins *et al*. discovered that 1 g/L iron shavings helped to get a COD removal of 65% in 120 min ozonation of olive mill wastewaters with an initial COD of 1211 mg/L^37^. Acid environment might help the iron corrosion, which leads to the formation of iron oxides or hydroxides and is beneficial for catalytic ozonation process. Nano-scale ZVI had a higher activity than large ZVI particle. However, the oxidation of nano-scale ZVI by O_2_ was too fast in air, and thus it should be stored in organic solvent, which limited its application in wastewater treatment.

### Catalytic ozonation performance under continuous mode

#### Effect of HRT

**Figure 3:**
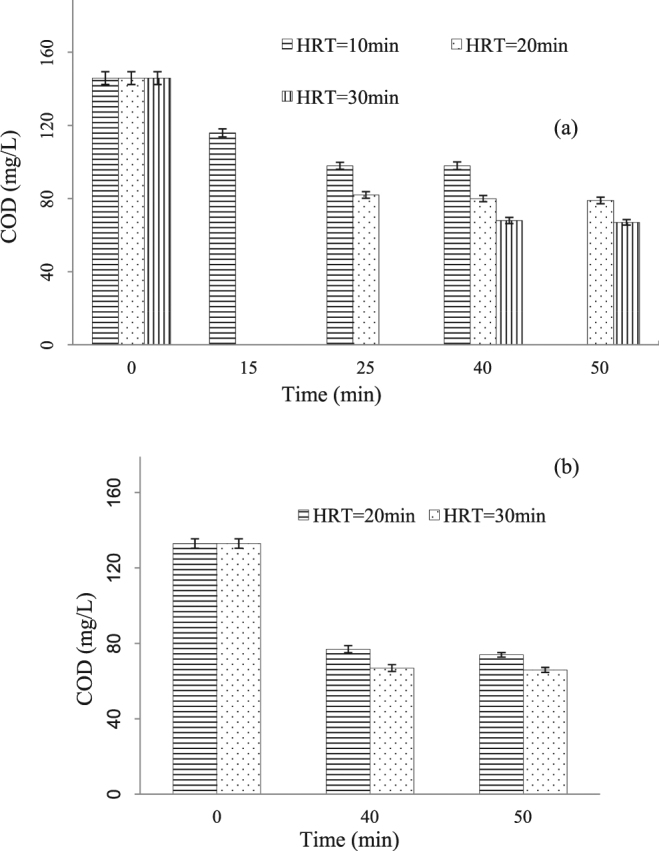
Effect of HRT on COD reduction during catalytic ozonation under continuous mode. Experimental conditions: (**a**) without addition of PAM; ozone dosage = 12.2 g-O_3_/min (ozone concentration = 120 mg/L; ozone flow rate = 6.08 m^3^/h); (**b**) with addition of 2 mg/L PAM; ozone dosage = 10.2 g-O_3_/min (ozone concentration = 120 mg/L; ozone flow rate = 5.11 m^3^/h).

From the current literatures, catalytic ozonation used in the degradation of organic pollutants were mostly in batch, semi-batch or fixed-bed reactors^35,38^. Batch reactor could be adopted in lab-scale application. Continuous mode appeared more frequently in pilot or full scale wastewater treatment processes because it is often simpler and more efficient. This part investigated the BDFW treatment by heterogeneous catalytic ozonation in continuous mode. Figure 3 displays the effects of different HRTs on COD removal, which shows that the effluent tends to stabilize when the sampling time is much longer than HRT. When the HRT was 10 min, the COD of the inlet BDFW decreased from the initial 146 mg/L to the final 98 mg/L, and the COD removal percentage kept stable after about 25 min running. When the HRT changed to 30 min, the COD of the effluent fell to 67 mg/L, and the corresponding COD removal percentage increased by 21% compared with that of the 10 min HRT. The ozone utilization efficiency can be calculated to be 1.13, 0.80 and 0.63 g COD/g ozone for HRT 10, 20 and 30 min, respectively. To meet the discharge standards, the HRT should be set at least 20 min.

To aid the pollutants removal, 2 mg/L of PAM - a well known organic polymer flocculants, was added to the heterogeneous catalytic ozonation reactor, at the same time, the wastewater in the reactor was recycled at a rate of 33.0 m^3^/h by a pump to accelerate the fluid mixture. However these measures did not achieve the expected better results. As shown in Fig. 3(b), when the HRT was 30 min, the COD decreased from 133 mg/L in the influent to 66 mg/L in the effluent, the ozone utilization efficiency was 0.64 gCOD/g ozone under this operation condition, the consequent improvement was just marginal. Internal recycling and PAM adding were both unnecessary due to the increasing cost for energy and chemical reagent.

#### Effect of ozone dosage

**Figure 4:**
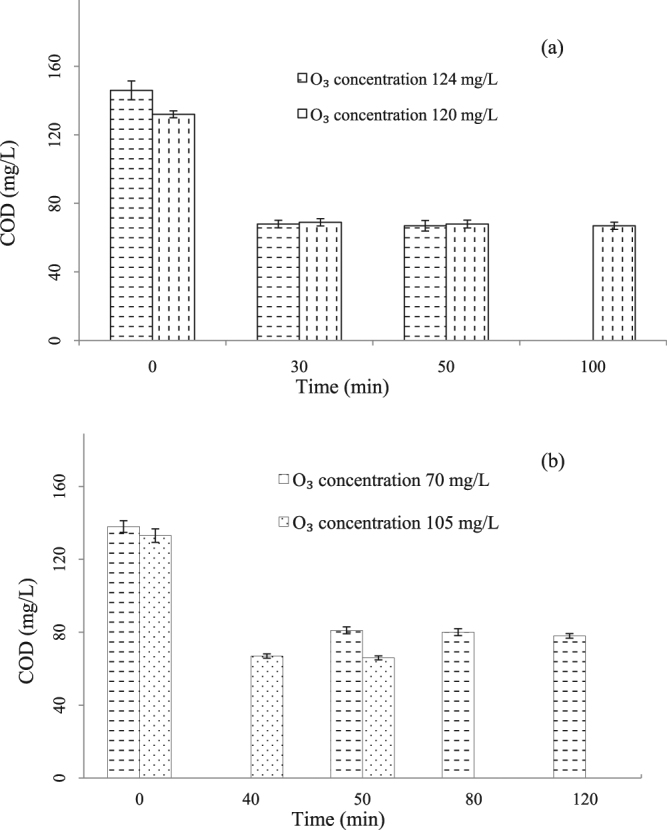
Effect of ozone dosage on COD reduction during catalytic ozonation under continuous mode. Experimental conditions: (**a**) without addition of PAM; HRT = 30 min; recycling rate = 0 m^3^/h; (**b**) with addition of PAM; HRT = 30 min; recycling rate = 33.0 m^3^/h.

The pilot scale heterogeneous catalytic ozonation of BDFW was investigated in terms of different ozone dosage. Several ozone concentrations were obtained by adjusting its gaseous concentration and gas flow rate. It seemed clear that higher ozone dosage could achieve better performance, just as the results indicated in Fig. 4. In section (a), under the operation condition of no PAM addition and no wastewater recycling, as the ozone dosage increased from 11.2 g-O_3_/min to 12.2 g-O_3_/min, the COD removal was enhanced from 49% to 54% consequently. Similar relationship between COD removal and ozone dosage was observed in the treatment of dyestuff wastewater by ceramic membrane separation coupled with catalytic ozonation^39^.

As the most important oxidant in heterogeneous catalytic ozonation system, higher ozone dosage provided more chances for active species generation, which was beneficial for pollutants degradation and caused deeper organic compounds removal. However, excessive ozone dosage is not always economical for the practical application. In the case of Fig. 4(b), the trend of increased ozone dosage brought increased COD removal did not alter at the presence of PAM as well as wastewater recycling. When the ozone dosage increased from 6.8 g-O_3_/min to 10.2 g-O_3_/min, the corresponding COD removal was enhanced from 44% to 50%. However, when the ozone utilization efficiency was inspected, it was noticed that the one with lower ozone dose was superior to the higher ozone dose with the ratio of 0.86 gCOD/g ozone over 0.64 gCOD/g ozone. Therefore after considering the treatment cost and the discharge requirements, a suitable ozone dose selection is necessary for the balance between pollutants removal and energy cost.

#### Effect of reflux ratio

**Figure 5:**
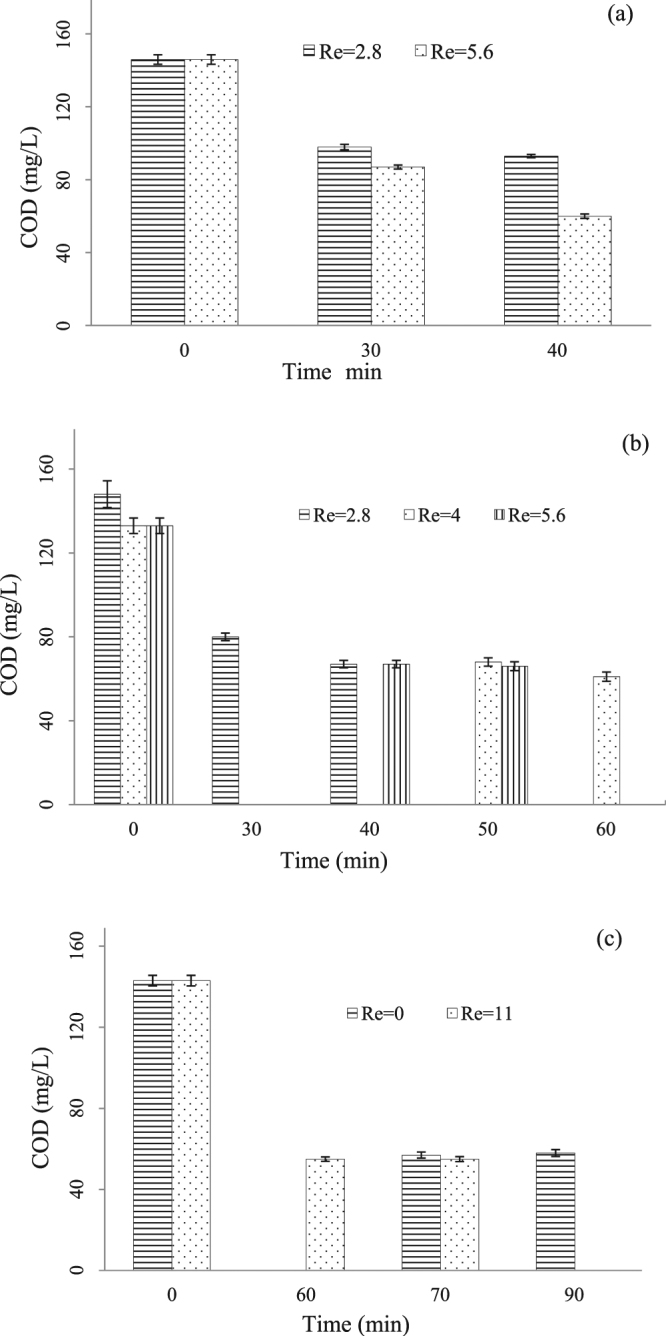
Effect of recycling rates on COD reduction during catalytic ozonation under continuous mode. Experimental conditions: (**a**) without addition of PAM; ozone dosage = 10.2 g-O_3_/min; HRT = 30 min; (**b**) with addition of PAM; ozone dosage = 10.2 g-O_3_/min; HRT = 30 min; (**c**) with addition of PAM; ozone dosage = 6.8 g-O_3_/min; HRT = 60 min.

In the continuous mode heterogeneous catalytic ozonation system, to promote the mixing of different materials with different phases, such as the dispersion of ozone gas in the wastewater, or the mixture among solid iron shavings and wastewater, a pump was used to circulate the wastewater in the reactor. Figure 5(a) shows the influence of wastewater recycling on the pollutant removal without the addition of PAM, where two reflux ratio of 2.8 and 5.6 were compared. The results revealed higher reflux ratio was advantageous to contaminants removal, the doubled reflux ratio brought about 23% of augment in COD removal. It was believed conventionally that the mixing assisted the migration of oxidants or active species across the interfacial between heterogeneous phases, therefore led to incremental reaction activity of the whole system, and provided more chances for pollutant-oxidant contact.

The results in Fig. 5(b,c) show that when PAM was added into the heterogeneous catalytic ozonation system, the impact of wastewater recycling on the COD removal turned to be unremarkable. For example, as the reflux ratios changed from 2.8 to 4.0, and to 5.6, the COD removal percentages just fluctuated around 51%. Even when the reflux ratio was raised from none to 5.6, the final COD removal percentage remained to be about 55%, while the rest test conditions were kept constant. This could be explained by two reasons. Firstly, Fe^3+^ was generated by the oxidation of waste iron shavings, and played as an effective *in situ* coagulant, which might cover the effect of PAM. Secondly, the main mechanism for organic removal was catalytic oxidation, and coagulation & flocculation process had less effect on COD reduction.

### Case study of catalytic ozonation by ZVI under suitable conditions

Table 1 shows the stable removal of contaminants by catalytic ozonation within a continuous running period of three months under the same conditions of 10.2 g-O_3_/min ozone dosage and flow rate of 5.8 m^3^/h, corresponded to a HRT of 30 min. The removal efficiencies of COD and sCOD were close to each other, which indicate that heterogeneous catalytic ozonation process was effective for both particles and soluble organic matters. The DOC removal efficiency was approximately 10% higher than the COD removal. Proteins and polysaccharides belong to typical SMPs; their removals were the highest, which indicated that SMPs were more vulnerable to degradation by heterogeneous catalytic ozonation. The removals of TN and TP were nearly negligible, thus heterogeneous catalytic ozonation is not applicable if TN or TP is the target pollutant. The inhibitory effect of BDFW dropped from 29% to 25%, implying that a small part of toxic pollutants were transformed to less toxic or non-toxic substances, thus manifested a slight reduction in the acute toxicity.

**Table 1:** Characteristics of the BDFW before and after heterogeneous catalytic ozonation.

	BDFW	Treated by O_3_/ZVI	Removal
pH	7.08 ± 0.20	7.33 ± 0.30	—
COD (mg/L)	165 ± 20	54 ± 5	67%
sCOD (mg/L)	93 ± 14	28 ± 4	70%
DOC (mg/L)	76 ± 6	16 ± 3	79%
Proteins (mg/L)	35 ± 5	7 ± 3	80%
Polysaccharides (mg/L)	10 ± 3	2 ± 1	85%
TN (mg/L)	56 ± 8	55 ± 7	—
TP (mg/L)	2.7 ± 0.2	2.6 ± 0.2	—
Inhibitory effect (%)	29 ± 3	25 ± 2	—

Experimental conditions: ozone dosage = 10.2 g-O_3_/min; HRT = 30 min; recycling rate = 0 m^3^/h.

The iron leaking during the heterogeneous catalytic ozonation was investigated, and the results in Table 2 demonstrate that the leaking iron was predominantly in solid form, namely ferric oxides and ferric hydroxides, or the corrosion products of iron during the ozonation process. Compared to the iron shavings dosage of 350 g/L, the iron leakage was minimal, and this indirectly implied that the iron shavings were of high stability as catalyst.

**Table 2:** Leaking of iron during catalytic ozonation of BDFW in continuous mode.

Sampling time (min)	Ozone dosage (g-O_3_/min)	HRT (min)	COD (mg/L)	Fe^2+^ (mg/L)	solid Fe (mg/L)	Total Fe (mg/L)
0	—	—	144	—	2.0	2.0
40	10.2	30	89	2.8	76	78.8

Experimental conditions: Recycling rate = 0 m^3^/h; HRT = 30 min; ozone dosage = 10.2 g-O_3_/min.

### Variance of organic compounds

**Figure 6:**
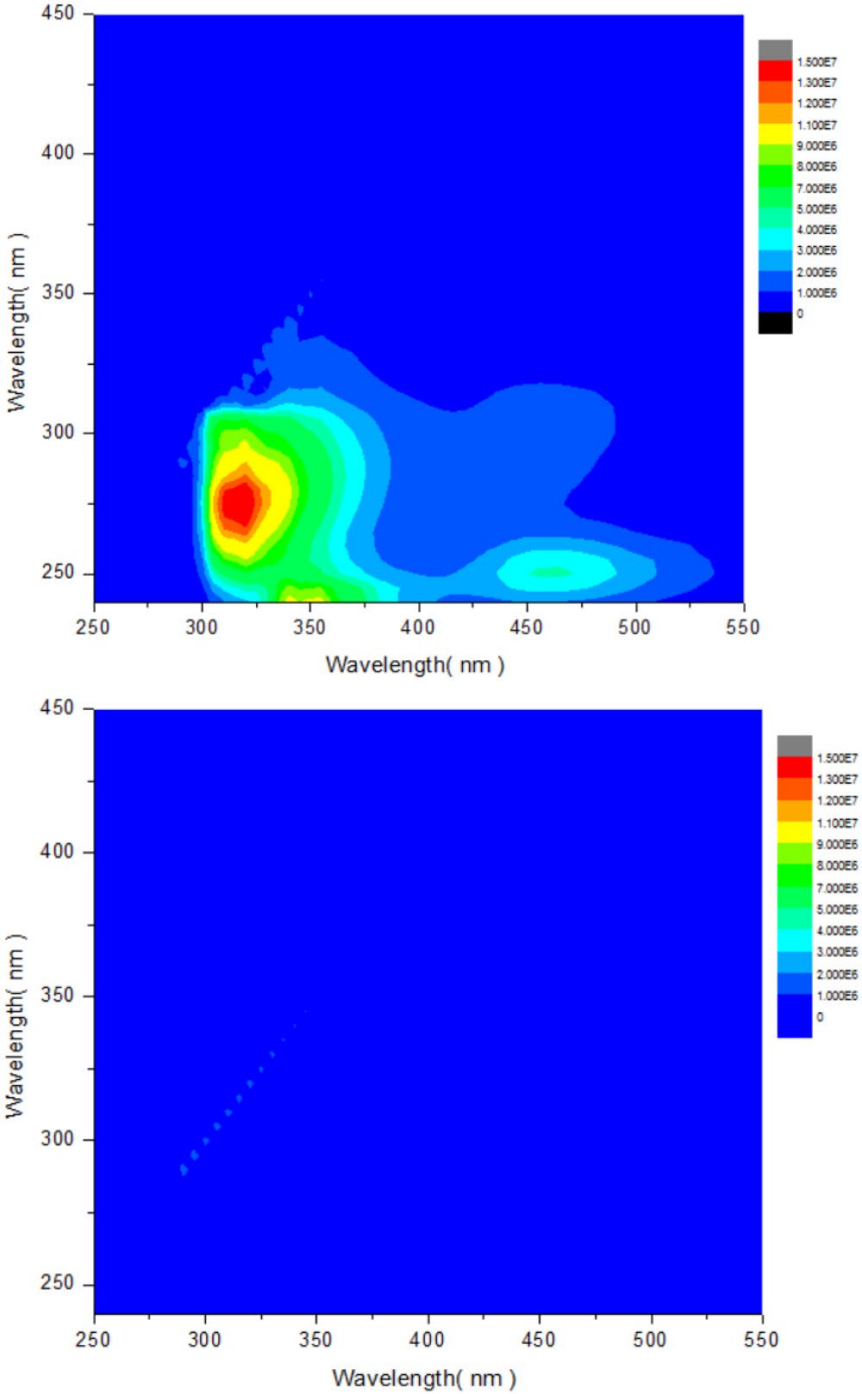
The removal of fluorescent substances in BDFW by heterogeneous catalytic ozonation. Experimental conditions: ozone dosage = 10.2 g-O_3_/min; HRT = 30 min; recycling rate = 0 m^3^/h.

Figure 6 shows the removal of fluorescent substances in BDFW with the assistance of 3DEEM fluorescence. There were 3 main peaks observed in BDFW representing protein-like (Ex/Em = 270/320 nm), aromatic protein (Ex/Em = 240/350 nm) and fulvic acid-like (Ex/Em = 250/460 nm) substance, respectively. The disappearance of protein-like peak was consistent with the 80.2% removal of proteins as shown in Table 1. Similar to Wu *et al*.^27^, all the 3 peaks disappeared after being treated by heterogeneous catalytic ozonation, indicating the strong capacity of this process for the removal of natural fluorescent substances.

Figure S1 and Table S1 show the variations in organic pollutant species before and after treatment by heterogeneous catalytic ozonation, it was discovered that 40 species of organic compounds were detected from the BDFW, 38 species of which were strong polar organics with retention time of ≤2 min in UHPLC-QTOF, this part comprised 74.7% of the total amount of organics in the BDFW regarding their peak area. The rest 2 were medium or weak polar organics with retention time of >2 min, and comprised 25.3% of the total organics amount. After treatment, the number of strong polar organics kept to be 38, but their share changed to be 93.1% in the effluent, the number of medium or weak polar organics was still 2, but their share decreased to 6.9% in the effluent. Among the 38 strong polar species, 20 of which were partly removed, and their peak area dropped by 16.1% compared to the original value. 18 of which were of lower molecular weight, their peak area increased by 36.6%, indicating the degradation of long chain molecule. For the 2 medium or weak polar organics, 1 of which was completely removed. Aside from that, there were 1 species increased in peak area and 1 species generated, which might be the degradation intermediate.

It is meaningful to identify the organics species in the wastewater before and after treatment to provide the information of how these molecules were degraded. Figure S2 and Table S2 demonstrate the organic compounds that could be detected by GC-MS before and after heterogeneous catalytic ozonation. It indicated that many long chain compounds were totally removed or partly removed, most of the textile auxiliaries (1-Tridecanamine, N,N-dimethyl-) were totally removed, and some chemicals with lower molecular weight (for example, Acetic acid, ethyl ester) were increased in amount or were newly generated as the degradation intermediates. For example, the generation of 1-Dodecanamine, N,N-dimethyl- might have some relationship with 1-Tridecanamine, N,N-dimethyl- with similar structures^27^. The removal of phenolic compounds could reduce the inhibitory effect as shown in Table 1. Moreover, catalytic ozonation could break down the azo bond, which reduced the toxicity of wastewater as well. Possible SMPs (hexadecanoic acid) and plasticizers (isooctyl phthalate) were significantly reduced after treatment. It is obviously that, besides polar compounds, catalytic ozonation with waste iron shavings also effectively removed non polar organic pollutants in pilot scale plant.

In summary, proteins (SMPs), aromatic protein and fulvic acid-like were effectively removed from secondary effluent by catalytic ozonation with ZVI. Moreover, significant reduction of the peaks for large molecules was observed together with the increase of the peaks for small molecules through HPLC-MS analysis for polar organic compounds. For the analysis of non polar organics, long chains of alkanes and esters with high molecular weight were reduced, leading to a decreased COD values. Mineralization of organics were also observed with a significantly decrease of DOC in the aqueous phase.

The ZVI is good electron donor with high surface activity, and it can be applied as a standalone technique for it is highly reactive towards some organic pollutants, economical, and less toxic^40^. The reactions between ZVI and H_2_O or H^+^ in the water generated nascent atom H, which could cleave the bonds, destroy the chromophore groups and conjugated system of large organic compounds^41,42^. The intermediate products such as Fe^2+^, Fe^3+^, and ferric hydroxides were thermodynamically active and unstable, the subsequently formed passive iron oxides layers like Fe_3_O_4_ or FeOOH were able to act as effective coagulants, or just worked as catalysts in ozonation^43,44^.

The individual application of ozone was common in the treatment of wastewater, and it has high efficiency in degrading compounds with functional groups that have high electron density such as aromatic compounds, through direct reaction^1^. *In situ* ·OH radical generation from the combined ozone/ZVI process has already been confirmed, owing to its high oxidizing potential (2.80 V) and non selectivity in nature. ·OH radicals were regarded as the main active species for the oxidation of organic pollutants in BDFW^27,45^. Therefore it could be concluded that different processes cooperated together to create synergetic effect on the removal of pollutants in the wastewater.

## Conclusion

The pilot scale heterogeneous catalytic ozonation plant was effective in the removal of organic pollutants from BDFW, which can meet the direct discharge limit of 80 mg/L of GB 4287–2012 under suitable conditions. The catalyst of iron shavings was stable and effective in property, economical in cost, thus is a good choice for heterogeneous catalytic ozonation. The results of analysis by UHPLC-QTOF and GC-MS revealed that many components in BDFW were effectively degraded, analyzing of the water quality data also indicated that the toxicity of the BDFW was reduced.

## Electronic supplementary material

Supplementary information
