# Supplementary material for: Pilot-scale study on catalytic ozonation of bio-treated dyeing and finishing wastewater using recycled waste iron shavings as a catalyst
Source: Sci Rep. 2018 May 15;8:7555. doi: 10.1038/s41598-018-25761-6 (PMC5954159; doi:10.1038/s41598-018-25761-6)
Supplement: Supplementary file 1 — Supplementary information [file 41598_2018_25761_MOESM1_ESM.docx]

**Supporting Information**

*for*

**Pilot-scale study on catalytic ozonation of bio-treated dyeing and finishing wastewater using recycled waste iron shavings as a catalyst**

Jieting Maa, Yunlu Chen^a^, Jianxin Nie^a^, Luming Mab, Yuanxing Huangc, Liang Li^c,^*, Yan Liu^a^, Zhigang Guo^a,^ **

*^a^* *Department of Environmental Science and Engineering, Fudan University, Shanghai, 200433, China;*

*^b^ College of Environmental Science and Engineering, Tongji University, Shanghai, 200092, China;*

*^c^ School of Environment and Architecture, University of Shanghai for Science and Technology, Shanghai, 200093, China;*

*^*^ Corresponding author: liliang@usst.edu.cn (L. Li)*

*^**^ Co-corresponding author: guozgg@fudan.edu.cn (Z. Guo)*

**Materials and methods**

The fluorescence spectra were tested with an excitation/emission wavelength of 250-450 & 250-550 nm using a fluorometer (HORIBA Jobin Yvon FluoroMax-4, France). The slit width for both excitation and emission was set at 5 nm, and the scan speed was 1200 nm/min. For UHPLC-QTOF analysis, the separation was performed on a UHPLC system (Model 1290, Agilent Corp., Milford, MA, USA) with an Agilent ZORBAX SB-C18 HD column (2.1 mm × 50 mm × 1.8 μm) at 30 °C. The analysis was performed with gradient elution using mobile phase (A) acetonitrile with 0.1% acetic acid and (B) ultrapure water with 0.1% acetic acid. The elution started with 5% A and 95% B for 2 mins, decreased to 100% A within 8 mins, kept for 5 mins, and returned initial conditions during 0.5 min, held for 4.5 mins. The flow rate and injection volume were set at 0.2 ml/min & 10 µL. Agilent 6540 QTOF mass spectrometer (Agilent Corp., Milford, MA, USA) was operated in both positive and negative electrospray ionization interface (ESI) mode. Ultrapure nitrogen (99.999%) was employed as drying and sheath gas with the same flow rate of 12 L/min but at different temperature of 325 & 400 ^o^C, respectively. The nebulizer pressure was set at 60 psi. The capillary and nozzle voltage were set at 0.5 and 2.0 keV, respectively. MS data were acquired over the m/z range of 50-3000 with a scan rate of 2 spectra/s.

**Figures and tables**

1. Figure S1 HPLC-MS chromatographs for the original wastewater and effluent treated by catalytic ozonation
2. Figure S2 GC-MS chromatographs for the original wastewater and effluent treated by catalytic ozonation
3. Table S1 The number of organic compounds before and after treatment as detected by UHPLC-QTOF
4. Table S2 Main organic compounds detected by GC-MS before and after heterogeneous catalytic ozonation

Figure S1 HPLC-MS chromatographs for the original wastewater and effluent treated by catalytic ozonation


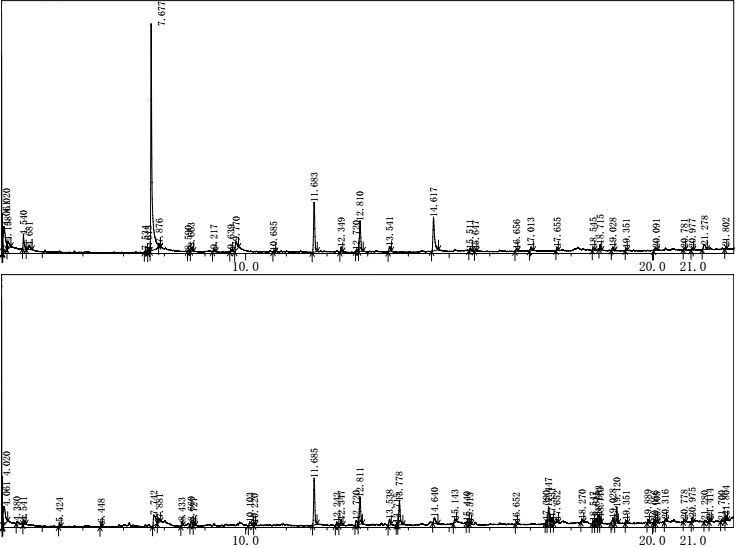


Figure S2 GC-MS chromatographs for the original wastewater and effluent treated by catalytic ozonation

**Table S1** The number of organic compounds before and after treatment as detected by UHPLC-QTOF

|  | Retention time ≤2 min | | Retention time ＞2 min | |
| --- | --- | --- | --- | --- |
|  | Number | Percentage (%) | Number | Percentage (%) |
| BDFW | 38 | 74.7^a^ | 2 | 25.3 |
| Treated effluent | 38 | 93.1^b^ | 2 | 6.9 |
|  |  |  |  |  |
| Variation |  |  |  |  |
| Totally removed | 0 | - | 1 | 23.9 |
| Partially removed | 20 | 16.1 | 0 | - |
| Increased | 18 | 36.6 | 1 | 0.7 |
| Newly generated | 0 | - | 1 | - |

Experimental conditions: ozone dosage=10.2 g-O_3_/min; HRT=30 min; recycling rate=0 m^3^/h;

^a^ peak area percentage in the total species of the BDFW

^b^ peak area percentage in the total species of the treated effluent

**Table S2** Main organic compounds detected by GC-MS before and after heterogeneous catalytic ozonation

| Organic compounds | Information (molecular formula; CAS; category) | BDFW |  | Treated |  |
| --- | --- | --- | --- | --- | --- |
|  |  | PA | RPA | PA | RPA |
| Totally removed |  |  |  |  |  |
| 2-Pentadecyl-1,3-dioxepane | C_20_H_40_O_2_; 41563-29-5 | 7.60E+04 | 1.3 |  |  |
| Propanoic acid, 3-hydroxy-2,2-dimethyl-, methyl ester | C_6_H_12_O_3_; 14002-80-3; Industrial chemicals | 4.86E+04 | 0.83 |  |  |
| 1,4,6,9-Tetraoxo-perhydropyridazo(1,2-a)pyridazine- | C_8_H_8_N_2_O_4_; 5343-01-1 | 3.43E+04 | 0.59 |  |  |
| 1-Tridecanamine, N,N-dimethyl- | C_15_H_33_N; 17373-29-4; Textile auxiliaries | 3.83E+05 | 6.55 |  |  |
| 1,7-Dimethyl-4-(1-methylethyl) cyclodecane | C_15_H_30_; 645-10-3 | 5.10E+04 | 0.87 |  |  |
| 1,2-Dichloro-3-nitrobenzene | C_6_H_3_Cl_2_NO_2_; 3209-22-1 | 1.06E+05 | 1.82 |  |  |
| 2H-1-Benzopyran-2-one, 7-methoxy- | C_10_H_8_O_3_; 531-59-9; Industrial chemicals | 2.13E+04 | 0.36 |  |  |
| Phthalic acid, bis(7-methyloctyl) ester | C_26_H_42_O_4_; 20548-62-3; Pharmaceuticals | 7.54E+04 | 1.29 |  |  |
| 2-Amino-5,6-dichloro-benzothiazole | C_7_H_4_Cl_2_N_2_S; 24072-75-1 Intermediates of Dyes and Pigments | 3.88E+04 | 0.66 |  |  |
| Benzene, 1,2,4-triethyl-5-methyl- | C_13_H_2_O; 19961-08-1 | 4.82E+05 | 8.24 |  |  |
| Octadecanoic acid | C_18_H_36_O_2_; 57-11-4; plasticizer, stabilizer and lubricant | 7.26E+04 | 1.24 |  |  |
| 1,3,5,7-Tetroxocane, 2,4,6,8-tetramethyl- | C_8_H_16_O_4_; 108-62-3; pesticide | 2.13E+04 | 0.36 |  |  |
| Ethanone, 1-(2,3,4-trimethylphenyl)- | C_11_H_14_O; 1467-36-3 | 4.84E+04 | 0.83 |  |  |
| 1,2,3,5-Benzenetetracarboxylic acid, tetramethyl ester | C_14_H_14_O_8_; 3034-97-7 | 2.84E+05 | 4.85 |  |  |
| Phenol, 2-(2-hydroxy-1,2-dimethylcyclopentyl)-, 1-acetate, trans- | C_15_H_20_O_3_; 39877-96-8 | 9.00E+04 | 1.54 |  |  |
| Phosphine, 1,3-propanediylbis[dicyclohexyl- | C_27_H_50_P_2_; 103099-52-1; Industrial chemicals | 3.98E+04 | 0.68 |  |  |
| 1,2,3,4-Benzenetetracarboxylic acid, tetramethyl ester | C_14_H_14_O_8_; 3451-02-3 | 1.27E+05 | 2.16 |  |  |
| Yohimban-16-carboxylic acid, 17-hydroxy-, methyl ester | C_21_H_26_N_2_O_3_; 146-48-5; Plant extracts | 2.17E+05 | 3.71 |  |  |
| Acetamidocyclohexane | C_8_H_15_NO; 1124-53-4 | 4.21E+04 | 0.72 |  |  |
| QUERCETIN 7,3',4'-TRIMETHOXY | C_18_H_16_O_7_; 6068-80-0 | 4.03E+04 | 0.69 |  |  |
| 2-Thiophenecarboxylic acid, 5-nonyl- | C_14_H_22_O_2_S; 59782-34-2 | 4.33E+04 | 0.74 |  |  |
| Partly removed |  |  |  |  |  |
| Phenol, 2,6-bis(1,1-dimethylethyl)-4-methyl- | C_15_H_24_O; 128-37-0; plastic additive | 9.72E+04 | 1.66 | 2.55E+04 | 0.24 |
| 7,9-Di-tert-butyl-1-oxaspiro(4,5)deca-6,9-diene-2,8-dione | C_17_H_24_O_3_; 82304-66-3 | 8.50E+04 | 1.45 | 7.68E+04 | 0.73 |
| Hexadecanoic acid | C_16_H_32_O_2_; 57-10-3; possible SMPs | 1.11E+05 | 1.9 | 4.55E+04 | 0.43 |
| 1-Cyclohexene-1-carboxylic acid, 4-(1,5-dimethyl-3-oxohexyl)-, methyl ester | C_16_H_26_O_3_; 26462-72-6 | 1.16E+05 | 1.98 | 1.15E+05 | 1.08 |
| Tetratriacontane | C_34_H_70_; 14167-59-0; Organic Building Blocks | 4.98E+04 | 0.85 | 4.48E+04 | 0.42 |
| Isooctyl phthalate | C_24_H_38_O_4_; 27554-26-3; Plasticizers | 9.85E+04 | 1.68 | 7.15E+04 | 0.68 |
| Heptadecane, 2,6,10,15-tetramethyl- | C_21_H_44_; 54833-48-6 | 3.31E+04 | 0.57 | 3.10E+04 | 0.29 |
| Octane, 2,3,3-trimethyl- | C_11_H_24_; 62016-30-2 | 2.51E+04 | 0.43 | 2.18E+04 | 0.21 |
| 1H-Purin-6-amine, [(2-fluorophenyl)methyl]- | C_12_H_10_FN_5_; 74421-44-6 | 7.84E+04 | 1.34 | 2.97E+04 | 0.28 |
| Increased |  |  |  |  |  |
| 1,6-Dideoxy-l-mannitol | C_6_H_14_O_6_; 68832-20-2 | 2.30E+05 | 3.93 | 2.54E+05 | 2.4 |
| Acetic acid, ethyl ester | C_4_H_8_O_2_; 141-78-6; organic solvent | 5.87E+05 | 10.02 | 9.37E+05 | 8.86 |
| Undecane | C_11_H_24_; 1120-21-4; Industrial chemicals | 5.96E+04 | 1.02 | 2.50E+05 | 2.36 |
| Propanoic acid, 2-methyl-, 1-(1,1-dimethylethyl)-2-methyl-1,3-propanediyl ester | C_16_H_30_O_4_; 74381-40-1 | 6.79E+04 | 1.16 | 7.99E+04 | 0.76 |
| Isobutyl phthalate | C_16_H_22_O_4_; 84-69-5; Plasticizer | 7.78E+05 | 13.28 | 9.84E+05 | 9.31 |
| Dibutyl phthalate | C_16_H_22_O_4_; 84-74-2; Plasticizer | 5.02E+05 | 8.57 | 6.56E+05 | 6.2 |
| Octane | C_8_H_18_; 111-65-9; organic solvent | 2.11E+04 | 0.36 | 3.43E+04 | 0.32 |
| Decane, 2,3,8-trimethyl- | C_13_H_28_; 62238-14-6 | 3.51E+04 | 0.6 | 4.39E+04 | 0.41 |
| Decane, 2,3,5,8-tetramethyl- | C_14_H_30_; 192823-15-7 | 3.75E+04 | 0.64 | 5.96E+04 | 0.56 |
| Newly generated |  |  |  |  |  |
| Hydrazinecarboxylic acid, phenylmethyl ester | C_8_H_10_N_2_O_2_; 5331-43-1 |  |  | 2.33E+05 | 2.2 |
| Benzene, (nitromethyl) | C_7_H_7_ NO_2_; 622-42-4 |  |  | 1.17E+05 | 1.1 |
| Ether, tert-butyl methyl | C_5_H_12_O; 1634-04-4; gasoline additive |  |  | 2.40E+04 | 0.23 |
| Hexane, 3,3-dimethyl- | C_8_H_18_; 563-16-6 |  |  | 3.31E+04 | 0.31 |
| 1-Dodecanamine, N,N-dimethyl- | C_14_H_31_N; 112-18-5; Textile auxiliaries |  |  | 4.68E+06 | 44.28 |
| Tetradecane | C_14_H_30_; 629-59-4 |  |  | 2.26E+04 | 0.21 |
| Nonane, 3,7-dimethyl- | C_11_H_24_; 17302-32-8 |  |  | 4.05E+04 | 0.38 |
| 1-Pentadecanamine, N,N-dimethyl- | C_17_H_37_N; 17678-60-3 |  |  | 2.48E+05 | 2.35 |
| 6-Methyl-1-octanol | C_9_H_20_O; 110453-78-6; Building Blocks for Liquid Crystals |  |  | 1.25E+04 | 0.12 |
| 1-Octanol, dimethyl- | C_10_H_22_O; 1333-49-9 |  |  | 2.49E+04 | 0.24 |
| N-Methyl-N-benzyltetradecanamine | C_22_H_39_N; 83690-72-6 |  |  | 9.02E+04 | 0.85 |

PA: peak area

RPA: relative peak area

Experimental conditions: ozone dosage=10.2 g-O_3_/min; HRT=30 min; recycling rate=0 m^3^/h;
